# Noise-robust fixation detection in eye movement data: Identification by two-means clustering (I2MC)

**DOI:** 10.3758/s13428-016-0822-1

**Published:** 2016-10-31

**Authors:** Roy S. Hessels, Diederick C. Niehorster, Chantal Kemner, Ignace T. C. Hooge

**Affiliations:** 1 Department of Experimental Psychology, Helmholtz Institute, Utrecht University, Utrecht, The Netherlands; 2 Department of Developmental Psychology, Utrecht University, Utrecht, The Netherlands; 3 Humanities Laboratory and Department of Psychology, Lund University, Lund, Sweden; 4 Institute for Psychology, University of Muenster, Muenster, Germany; 5 Brain Center Rudolf Magnus, University Medical Centre Utrecht, Utrecht, The Netherlands

**Keywords:** Eye-tracking, Fixation detection, Noise, Data quality, Data loss

## Abstract

Eye-tracking research in infants and older children has gained a lot of momentum over the last decades. Although eye-tracking research in these participant groups has become easier with the advance of the remote eye-tracker, this often comes at the cost of poorer data quality than in research with well-trained adults (Hessels, Andersson, Hooge, Nyström, & Kemner *Infancy*, *20*, 601–633, 2015; Wass, Forssman, & Leppänen *Infancy*, *19*, 427–460, 2014). Current fixation detection algorithms are not built for data from infants and young children. As a result, some researchers have even turned to hand correction of fixation detections (Saez de Urabain, Johnson, & Smith *Behavior Research Methods*, *47*, 53–72, 2015). Here we introduce a fixation detection algorithm—identification by two-means clustering (I2MC)—built specifically for data across a wide range of noise levels and when periods of data loss may occur. We evaluated the I2MC algorithm against seven state-of-the-art event detection algorithms, and report that the I2MC algorithm’s output is the most robust to high noise and data loss levels. The algorithm is automatic, works offline, and is suitable for eye-tracking data recorded with remote or tower-mounted eye-trackers using static stimuli. In addition to application of the I2MC algorithm in eye-tracking research with infants, school children, and certain patient groups, the I2MC algorithm also may be useful when the noise and data loss levels are markedly different between trials, participants, or time points (e.g., longitudinal research).

The emergence of the remote video-based eye-tracker has allowed researchers to conduct eye movement research with a plethora of participant groups for which conventional eye-tracking techniques are unsuitable. Unlike, for example, scleral coil techniques or head-mounted and tower-mounted video-based eye-trackers, remote eye-trackers can be positioned at a distance from the participants and allow them to move freely within a specified range. Remote video-based eye-trackers are therefore suitable to use in participant groups whose head movements are difficult to restrain, such as infants (Oakes, 2012) or school children (e.g., Holmberg, Holmqvist, & Sandberg, 2015). As a result, eye-tracking research in, for instance, infants has gained a lot of momentum over the last decades (e.g., Aslin & McMurray, 2004; Oakes, 2012). Although eye-tracking research in these participants groups has become easier with the advance of remote eye-trackers, and although studies are available that provide advice on how to choose an eye-tracker for research in nonoptimal conditions (Hessels, Cornelissen, Kemner, & Hooge, 2015), data quality is still often low relative to recordings of well-trained adults (Hessels, Andersson, Hooge, Nyström, & Kemner, 2015; Wass, Forssman, & Leppänen, 2014; Wass, Smith, & Johnson, 2013). Current solutions for the automatic detection of one of the most commonly investigated events, fixations, in eye-tracking data are not built for low-quality data. This applies to both the solutions provided by eye-tracker manufacturers and the research community. This is problematic for eye-tracking research with infants and young children, in which data of low quality frequently occur. As a result, some researchers have moved away from fully automatic analysis techniques and turned to manual correction of fixation detection in eye movement data from infants (Saez de Urabain, Johnson, & Smith, 2015). Here we consider the consequences of low data quality for fixation detection, describe and quantify the noise in infant data from remote video-based eye-trackers, and introduce a new and superior solution for detecting fixations in noisy data.

In humans, visual acuity is greatest at the fovea, and eye movements are made to bring an area of the visual scene onto the fovea, or to maintain it there. There is a primary distinction between the periods in which an area of the visual scene is kept on the fovea—a fixation—and periods in which an area of the visual scene is brought onto the fovea—a rapid eye position change called a saccade. Figure 1 (top panel) depicts typical eye movement data from an eye-tracker using static stimuli. At first glance, the periods in which gaze position is constant—the fixations—can clearly be discriminated from the periods of rapid gaze position change—the saccades. Labeling segments of the eye movement data as fixations and saccades may give researchers insight into the spatiotemporal processing of a visual scene. Algorithms that label eye movement data in this fashion are referred to as *event detection* algorithms, where an event can be a fixation, smooth pursuit (when using moving stimuli; e.g., Larsson, Nyström, Andersson, & Stridh, 2015), saccade, blink, postsaccadic oscillation (see, e.g., Nyström, Hooge, & Holmqvist, 2013), and so forth. In the present article, we focus on the labeling of fixations in data from remote or tower-mounted eye-trackers using static stimuli. More specifically, we investigated fixation labeling under varying levels of noise in the eye movement data, to mimic fixation detection in low- and high-quality eye movement data.

**Fig. 1:**
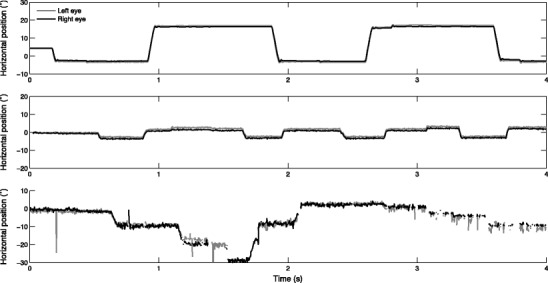
Example eye-tracking data. The top graph depicts data recorded at 500 Hz on the SR Research EyeLink 1000 from an adult participant by Hooge et al. (2015). The middle graph depicts data recorded at 300 Hz on the Tobii TX300 from an adult participant by Hessels, Kemner, et al. (2015). The bottom graph depicts data recorded at 300 Hz on the Tobii TX300 from an infant participant by Hessels, Andersson, et al. (2015). Only the horizontal coordinates are shown; the middle of the screen is at 0°

## Event detection and data quality

An event detection algorithm generally consists of two parts. The first, which we refer to as the “search rule,” aims to separate fast periods (saccades) and slow periods (fixations) in the data from each other. The second part, the “categorization rule(s),” accepts, rejects, and/or merges the saccade and/or fixation candidates from the first rule according to a set of criteria. These criteria may include, for instance, a minimum fixation time, maximum saccade duration, and so forth. Moreover, these criteria may be based on physiological constraints of the eye—for example, a maximum acceleration during a saccade (Nyström & Holmqvist, 2010)—or on the experimental setup—for example, a minimum saccade amplitude of 2° when the elements to be fixated are spaced 4° apart. Event detection algorithms are often referred to by their search rule. For example, two popular types of event detection algorithms for labeling fixations and saccades are *velocity*- and *dispersion*-based algorithms (see Holmqvist et al., 2011, pp. 147–175, for an elaborate overview). Velocity-based event detection algorithms compute a velocity signal from the gaze position signal, and subsequently use a velocity cutoff to label periods of data as fixation candidates. The velocity cutoff used may be set in advance, for example at 30°/s. Other strategies involve first detecting saccade candidates with a fixed threshold, and subsequently finding fixation start and end points by adapting the threshold to the mean velocity in a period preceding the saccade candidate (Smeets & Hooge, 2003) or using the median velocity plus a certain number of standard deviations (Engbert & Kliegl, 2003). Dispersion-based algorithms, on the other hand, label periods of data as “fixation candidates” when a set of subsequent samples exceed a minimum time and do not exceed a maximum distance from each other. As long as subsequent samples stay within the distance limit, they are added to the fixation candidate. If, however, a subsequent sample does exceed the maximum distance, the current fixation candidate is ended and a new fixation candidate is started in the next available window that fits the minimum-time and maximum-distance requirements. Both event detection algorithms may subsequently employ different or identical categorization rules to accept, reject, or merge fixation candidates into fixations.

The two classes of event detection algorithms just discussed are widely applied for analyzing eye movement data. Although such event detection algorithms may lead to reasonable results for adult data with large saccades and a low noise level (Fig. 1, top panel), they may not necessarily do so for data with a greater noise level. Figure 1 (middle panel) depicts adult data in which the noise level is higher and smaller saccades were made. However, the fixations and saccades are still relatively easy to distinguish at first glance. In infant data (Fig. 1, bottom panel), the amplitude of noise is frequently higher than in adult data, and often over short bursts no data are reported by the eye-tracker (Hessels, Andersson, et al., 2015; Wass et al., 2014). How these differences in data quality affect event detection may depend on the specific event detection algorithm used.

Two aspects of data quality are important to consider for the present event detection purposes: spatial precision and data loss.^1^ First, the *spatial precision* of the data refers to the reliability of the measurement when no movement of the eye takes place: the variable error, or noise, in the signal. Although the eye always moves slightly (e.g., tremor or drift), one way to estimate the noise amplitude is to calculate the sample-to-sample position change during fixation (Holmqvist, Nyström, & Mulvey, 2012). When the sample-to-sample position change is low, so is the noise amplitude. One may observe that the noise amplitude is lowest for adult data using the SR Research EyeLink 1000 (Fig. 1, top panel), followed by adult data using the Tobii TX300 (Fig. 1, middle panel), and finally for infant data using the Tobii TX300 (Fig. 1, bottom panel). The noise level is determined not only by the hardware (i.e., the eye-tracker), but also by the behavior of the participant group (Holmqvist et al., 2011). For instance, the poorer data quality in infant eye-tracking research may in part be due to the higher amount of movement in infants. An increase in noise amplitude may affect outcome measures such as the number of fixation candidates and the mean duration of fixation candidates. Moreover, depending on the specific search rule used, the number of fixation candidates and the fixation duration may either increase or decrease. If a fixed velocity threshold is used to separate fixations from saccades, decreased precision may break up long fixation candidates into shorter fixation candidates due to noise spuriously exceeding the threshold (Wass et al., 2013). When long fixation candidates are broken up into multiple shorter fixation candidates, the number of fixations increases, and the mean fixation duration decreases. On the other hand, if a velocity threshold is adaptively chosen on the basis of the noise amplitude in the data, small saccades with a velocity close to that of the noise may be missed. This may then cause multiple fixation candidates to be merged into longer fixation candidates (Holmqvist et al., 2012). As a consequence, the number of fixations decreases, and the mean fixation duration increases.

The second aspect of data quality, *data loss*, refers to periods in which no position coordinates are reported by the eye-tracker. Although this intuitively may be attributed to a participant not looking at the screen, data loss may often occur due to unstable tracking of the eye by the eye-tracker (Hessels, Andersson, et al., 2015; Wass et al., 2014). Figure 1 (bottom panel) depicts such brief loss of contact: Between 3 and 4 s, the recorded data are interrupted by short periods of data loss. During event detection, fixation candidates may be broken up by periods of data loss (Holmqvist et al., 2012). When more data loss occurs, the number of fixations increases, and the mean fixation duration decreases, as compared to lower data loss levels. Whether changing the parameters of the categorization rule(s) may compensate for the differences in output by the search rule is part of ongoing research (Zemblys & Holmqvist, 2016). On the other hand, although the problem of reduced data quality in, for instance, infant research is a common one, few solutions have been designed to accommodate it.

## Event detection in noisy data

To our knowledge, two solutions have been proposed specifically to accomplish event detection in noisy data (Saez de Urabain et al., 2015; Wass et al., 2013). Wass et al. adapted a velocity-based event detection algorithm specifically designed to cope with eye movement data from infants. The search rule of this algorithm is as follows: The algorithm first selects only the portions for which data for both eyes are available, and applies a smoothing procedure. Subsequently, periods of data loss up to 150 ms are interpolated if the velocity between the start and end of the data loss period does not exceed the velocity threshold. Thereafter, all periods of data below the velocity threshold of 35°/s are marked as fixation candidates. The categorization rules that follow to label fixation candidates as fixations are extensive. First, if a fixation candidate borders on a period of data loss, it is excluded. Second, saccades are excluded (and fixation candidates consequently merged) if the fixation candidates before and after are within 0.25° distance from each other. Third, if a saccade is preceded by a fixation candidate with an average velocity over 12°/s, the saccade and the bordering fixation candidate are excluded. Fourth, if a saccade is preceded by three samples with an average velocity over 12°/s, the saccade and bordering fixation candidate are also excluded. Fifth, if the distance between the gaze positions for the two eyes prior to the saccade is larger than 3.6°, the saccade and bordering fixation candidates are excluded. Finally, fixation candidates shorter than 100 ms are excluded. Wass et al. reported that this algorithm remains reliable for data containing higher noise amplitude, whereas standard dispersion-based algorithms decrease in reliability with increasing noise amplitude. Saez de Urabain et al., on the other hand, use a two-step approach to event detection. In a graphical user interface, the user can set a number of parameters for a first estimation of which data segments are fixation candidates (i.e., the search rule). Thereafter, the user may manually correct the fixation candidates (i.e., a manual categorization procedure). Although the machine-coding, manual-correction approach by Saez de Urabain et al. may increase the amount of eye movement data that can be successfully labeled as fixations and used in further analysis, it is a highly time-consuming, and subjective, process. Moreover, although the velocity threshold adaptation by Wass et al. is automatic, it features a large number of categorization rules for rejecting data, leading to significant amounts of data being excluded when the noise level is high. Intuitively, an ideal approach would be an algorithm that is automatic and that can reliably achieve fixation labeling in periods of noisy data, instead of excluding such data. Here we introduce such an approach.

The present article introduces the “identification by two-means clustering” (I2MC) algorithm. This algorithm was specifically designed to accomplish the labeling of fixations across a wide range of noise levels and when periods of data loss may be present, without the need to set a large number of parameters or perform manual coding. Before we introduce how the algorithm operates, we first discuss how to the algorithm is to be evaluated.

## Evaluating the algorithm

How can the output of an event detection algorithm be evaluated? Or, the question that more generally arises; is the output from my event detection algorithm “correct”? Intuitively, it makes sense to ask this question. One would want to know whether the fixations labeled by an algorithm are “correct,” or whether the output of the algorithm conforms to a golden standard. The problem is, however, that although researchers appear to informally discuss fixations and saccades with relative ease, there is no gold standard for when a fixation starts or stops (Andersson, Larsson, Holmqvist, Stridh, & Nyström, 2016). Essentially, a fixation is defined by how it is computed, which differs for each event detection algorithm. Holmqvist et al., (2011, p. 150) also note “In reality, perfect matches between the fixations detected by an algorithm and moments of stillness of the eye are very rare. To make matters worse, the term fixation is sometimes also used for the period during which the fixated entity is cognitively processed by the participant. The oculomotor, the algorithmically detected, and the cognitive ‘fixations’ largely overlap, but are not the same.” Tackling the evaluation of algorithms based on the fixations detected is therefore problematic, as the definitions for fixations differ between algorithms.

Komogortsev, Gobert, Jayarathna, Koh, and Gowda (2010), for instance, aimed to determine a goodness of fit of the eye movement data to the stimulus that was presented. Participants were presented with a white dot that appeared sequentially at 15 locations on screen for one second each. In this case, an event detection algorithm was considered ideal if it detects 15 fixations, 14 saccades, an average fixation duration of one second, and an accuracy of 0°. Importantly, Komogortsev et al. (2010) noted that this method is inherently flawed. For instance, eye-trackers typically report accuracies of 0.5°, even under ideal circumstances. Moreover, 14 saccades and 15 fixations would imply that no corrective saccades are made. However, Komogortsev et al. (2010) preferred their method over manual techniques, which “are frequently employed to classify eye-movement behavior. However, this type of classification technique [i.e., manual coding] is susceptible to human error and can be open for biased interpretation with limited generalizability” (p. 2643). Andersson et al. (2016), on the other hand, employed two experienced human coders as their gold standard for comparing with algorithms. They noted that although human coders generally agreed more with each other than with automatic algorithms, there is a problem: Human coders make mistakes and have disagreements, and eventually it is impossible to determine whether an algorithm or a human coder should be “right.” Finally, in the approach by Zemblys and Holmqvist (2016), the algorithm considered “best” by the authors was taken as the gold standard—a choice that will most likely provoke debate. To sum, there is little consensus on the manner of evaluating the performance of a fixation detection algorithm.

Instead of focusing on whether a gold standard for the evaluation of fixation detection algorithms can be approximated, we take a different approach here. The purpose of the presented algorithm, called *I2MC*, is to achieve consistent labeling of fixations when there may be large differences in data quality between participants and between trials, as is often encountered in, for instance, infant research. This means that the I2MC algorithm should achieve fixation labeling across a range of noise amplitudes and when short periods of data loss may be present. If the labeling of fixations is done for data with a small noise amplitude and few periods of data loss, the output thereof can be compared to the output after adding noise of higher amplitude and periods of data loss to the same data. If the number of labeled fixations and the corresponding distribution of fixation durations remain unchanged as noise and data loss increase, the algorithm is considered to be robust to noise and data loss. The key decision left to the eye movement researcher is then whether the fixation-labeling output at low noise and data loss levels is satisfactory. If one is satisfied with the initial output of the algorithm, one can generalize this satisfaction to higher noise and data loss levels, given the algorithm’s stability in the face of increasing noise and data loss. This decision inevitably has to be made by every researcher, since no two experimental setups and data sets are identical.

To evaluate whether the I2MC algorithm improves on the currently available solutions, seven competing state-of-the-art algorithms were chosen from the literature. The general motivation for including an algorithm is that it should be able to deal with one or more of the data quality issues that we outlined above. The specific motivations are given in the Method section. As we discussed briefly, algorithms may differ in both their search rules and their categorization rules for labeling fixations. Moreover, the preprocessing steps, such as data smoothing and interpolation to impute missing data, prior to application of these rules may differ between algorithms. It is paramount to note that the focus here is not on finding a combination of preprocessing steps with search and categorization rules that produces the most noise-robust output by testing all possible combinations and exhaustively searching through their parameter spaces. Instead, the focus is on whether previous solutions, taken *as is*, produce noise-robust output, and whether or not the present I2MC algorithm improves over them. We reasoned that taking the algorithms as they come “out of the box” is what the vast majority of researchers who are not experts on event detection algorithms would do when choosing algorithms for purposes of data analysis.

Because previous research has shown that increased noise amplitude may affect the number of fixations, and consequently the fixation durations (Holmqvist et al., 2012; Wass et al., 2013), we calculated the numbers of fixations, mean fixation durations, and standard deviations of the fixation durations for all noise and data loss levels. Such an approach is similar to that of Zemblys and Holmqvist (2016), who investigated the parameter settings of event detection algorithms as a function of increasing noise level. However, their aim was to see how the settings of an algorithm should be adapted for the output to approach that of their gold standard (the algorithm they considered to be the “best”). Here, however, we compared each algorithm against itself to examine its robustness of the outcome measures to noise and data loss levels. After examining the noise robustness of the outcome measures of the I2MC algorithm in this manner, we examined the application of the I2MC algorithm to infant data.

## Algorithm

**Fig. 2:**
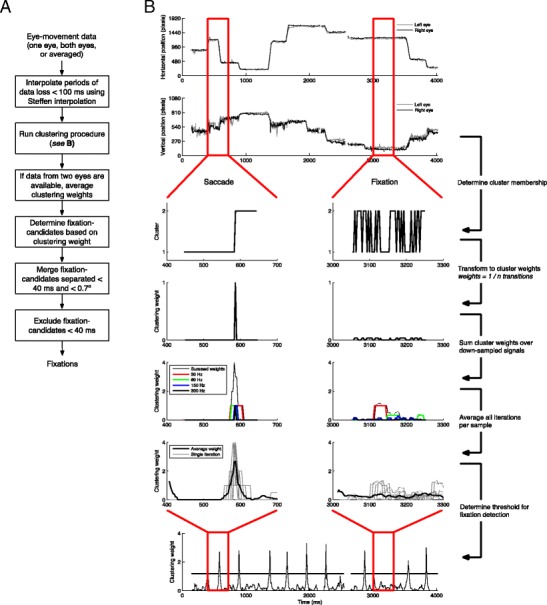
Overview of the I2MC algorithm: (A) flow-chart for the entire algorithm, and (B) the specific steps of the clustering procedure (as outlined in the Two-Means Clustering section)

The I2MC algorithm is composed of three separate steps: interpolation of missing data, two-means clustering (i.e., the selection of fixation candidates by the search rule), and finally fixation labeling (the categorization rules). It is important to note that although example values will be provided with the algorithm for all parameters, along with a motivation for the specific value, these values may need to be adapted to better suit a specific data set. A flowchart for the algorithm is depicted in panel A of Fig. 2. A MATLAB implementation of the I2MC algorithm is freely available from http://dx.doi.org/10.5281/zenodo.159456.

### Interpolation of missing data

To maximize the amount of eye-tracking data that can be used for event detection, imputation of short periods of missing data is performed through interpolation. We chose an interpolation method satisfying two conditions: (1) interpolation must be locally determined by the gaze samples at each end of the interpolation window, and (2) interpolation must be monotonic (i.e., there are no extrema in the data points between the start and end points). The interpolation method adopted here, which satisfies both constraints, was developed by Steffen (1990). It should be noted that the commonly used cubic spline interpolation (e.g., Frank, Vul, & Johnson, 2009) does not satisfy the constraints posed here, because it can produce extrema in the interpolated data.

Interpolation was performed as follows. Periods of missing coordinates in the gaze coordinate signals were interpolated provided that the following criteria were met. First, the period of missing coordinates had to be shorter than a set value. The value used in this article was 100 ms, and was chosen so as not to interpolate over entire saccade–fixation–saccade sequences (saccades with a latency of 100 ms are considered extremely early; Fischer & Ramsperger, 1984). In addition, blink durations are usually higher than 100 ms, and so relatively few blinks should be interpolated. The value of 100 ms is, however, not fixed, and may be adapted according to the periods of data loss observed in the eye-tracking data. Second, valid data had to be available for at least two samples at each end of the missing window.

### Two-means clustering

Following interpolation, a moving window of 200 ms width slides over the gaze position signal. The value of 200 ms was chosen here so that a window generally would contain parts of at most two, and no more, fixations. For each window, a two-means clustering procedure is carried out. Two-means clustering is a variant of *k*-means clustering (where *k =* 2 in this case), a procedure in which a number of observations are clustered iteratively into *k* clusters (see, e.g., Jain, 2010). The observations belonging to each cluster are those that are closer to the mean of that cluster’s observations than to the mean of any other cluster. In the present application, portions of the gaze position signal within a moving window are forced into two clusters. The overarching idea is that *if* the gaze position signal in a given window contains a saccade, there will be few cluster membership transitions, and these will be concentrated around a specific point in time—the time point of the saccade. *If*, however, the gaze position signal in a given window contains only a fixation, the cluster membership transitions between the two clusters are driven only by the noise in the fixations. They may thus occur frequently and are likely to be spread out across the whole window. The specific algorithmic steps of the clustering procedure, which are depicted in Fig. 2, panel B, are as follows:

1. If the current window contains no missing data (if it does, go to Step 4): Force the gaze position signal into two clusters. Cluster membership is a value of either 1 or 2 (i.e., the cluster the sample belongs to). It is important to note that cluster membership itself is not relevant, but only where the membership transitions occur from Cluster 1 to Cluster 2, or vice versa. Only the times at which these transitions from one cluster to another occur are used in the next step.
2. Construct a *clustering weight* for the current window from the cluster membership determined in Step 1. The clustering weight for samples in which a cluster membership transition occurs is *1/number of total transitions in the window*. The clustering weight for the other samples (i.e., those at which no transition occurs) is *0*. If, for example, one transition occurs from Cluster 1 to Cluster 2, as in the saccade example in Fig. 2, the clustering weight for the sample containing the transition is 1. For all samples containing a transition in the fixation example in Fig. 2, the clustering weight is much lower, because there are many transitions from one cluster to the other in the window.
3. To ensure that transitions are not caused solely by high-frequency noise in the data, down-sample the gaze position signal to integer divisions of the original sampling frequency, and repeat Steps 1 and 2 for each down-sampled position signal. For example, the data in Fig. 2 (and in this article) were recorded at 300 Hz and down-sampled to 150, 60, and 30 Hz. The clustering weights for the gaze position signal at its original sampling frequency, as well as the down-sampled signals, are subsequently summed.
4. Move the window in the gaze position signal. The window may be moved either one sample or a number of samples. We used a step of 20 ms (six samples at 300 Hz) here, because it provides nearly identical results to moving the window one sample, but decreases the computation time sixfold. This step size is, however, configurable. If the subsequent window contains missing data or is moved past the end of the data, go back in steps of one sample to determine the last possible window. If no additional windows are possible backward in time up to the previous possible window, find the first possible window after the period of missing data. As long as the end of the window does not reach the end of the gaze position signal, return to Step 1.
5. For each sample, average the clustering weights assigned in Steps 1–3 for each time that sample was included in the moving window. For example, if a sample was included in three windows, it will have been assigned three clustering weights, and these three weights are averaged. The subsequent clustering-weight signal (see Fig. 2) can now be used for fixation detection.

For binocular eye-tracking data, the clustering procedure described above was run on the data for the left and right eyes separately. These two clustering-weight signals were then averaged to determine the final clustering-weight signal. Doing this has the advantage that if only one eye moved according to the eye-tracker, which most likely represents noise, the result is unlikely to lead to a large peak in the clustering-weight signal. For monocular eye-tracking data, and when the data from only one eye are available in binocular eye-tracking data due to data loss, the clustering-weight signal from that one eye is used.

### Fixation labeling

The categorization rules for the present algorithm are as follows. A cutoff is used to determine fixation candidates from the clustering-weight signal (see panel B in Fig. 2). Here we used a cutoff of the mean clustering weight plus two standard deviations. However, different cutoffs may be required for different datasets. All periods of clustering-weight signal below this cutoff are labeled as *fixation candidates*, and thereafter consecutive fixation candidates are merged. Finally, short fixation candidates are excluded from the output. The settings for merging fixation candidates may depend on the stimuli used in the experiment, the noise level in the eye-tracking data, or the size of the saccades of interest. Here we opted for merging fixation candidates that were less than 0.7° apart and were separated by less than 40 ms. Fixation candidates shorter than 40 ms were removed.

The options that may be set in the algorithm and their suggested values are summarized in Table 1.

**Table 1:** Settings for the identification by two-means clustering (I2MC) algorithm and their suggested values for data at 300 Hz

Setting	Used Value(s) at 300 Hz	Impact When Changed
Interpolation window	100 ms	Increase will lead to interpolation of blinks, decrease will lead to less periods of data loss being interpolated.
Interpolation edge	6.7 ms (two samples)	Increase will require more data points at data loss edge, and will not interpolate in the event of flicker (i.e., repetition of short period of data loss and data points). At least two samples are required.
Clustering window size	200 ms	Increase will lead to clustering procedure being more readily carried out over saccade-fixation-saccade sequence.
Downsampling	150, 60, and 30 Hz	Removal of downsampling steps will lead to more susceptibility to short bursts of noise.
Window step size	20 ms	We observed no difference between 3.3 and 20 ms.
Clustering-weight cutoff	2 standard deviations above the mean	Increase will lead to fewer fixation candidates (more conservative), decrease to more fixation candidates (more liberal).
Merge fixation distance	0.7°	Increase will lead to more fixation candidates being merged.
Merge fixation time	40 ms	Increase will lead to more fixation candidates being merged.
Min. fixation duration	40 ms	Increase will lead to more short fixation candidates being excluded.

## Method

The algorithms were compared in terms of the following outcome measures: the number of fixations, the mean fixation duration, and the standard deviation of the fixation duration. These outcome measures were obtained from eye movement data with increasing noise amplitude and periods of data loss, as well as the combination of the two. A dataset consisting of binocular data that were recorded with the SR Research EyeLink 1000 by Hooge, Nyström, Cornelissen, and Holmqvist (2015) was used, in which the noise amplitude and data loss were artificially increased. This dataset was chosen on the basis of its low noise amplitude and low data loss levels. In Hooge et al.’s experiment, the participants made horizontal and vertical saccades of a wide range of amplitudes.

To examine robustness, the noise amplitude and data loss level in the data were artificially increased. To provide a valid test of algorithm performance, it is important that the added noise and data loss be representative of levels that actually occur when doing eye-tracking research in suboptimal conditions. To achieve this, we first characterized what the noise and data loss looked like in a set of infant data recorded by Hessels, Andersson, et al. (2015; for an example, see the bottom panel of Fig. 1). We subsequently developed methods to add noise and data loss with these characteristics at varying levels to our clean data. These methods are described in detail in Appendix A. The noise level was varied from a sample-to-sample root-mean square (RMS) noise level of 0 to 5.57°. The latter value was chosen as being beyond the upper limit for noise typically encountered in eye movement data. For comparison, the RMS noise level in infant eye movement data rarely exceeds 3° (Hessels, Kemner, van den Boomen, & Hooge, 2015). Data loss was varied by changing the occurrence of periods of data loss from 0 % to 100 % of a trial. Because the noise and data loss were characterized in a dataset recorded at 300 Hz, our clean data to which we subsequently added noise and data loss were down-sampled from 1000 to 300 Hz using first-order interpolation.

After examining the noise robustness of I2MC and competing algorithms in eye movement data with a range of artificially generated noise and data loss levels, we applied the algorithms to real infant data. To interpret the outcome measures of the algorithms when applied to the infant data, four eye movement experts (authors R.H., D.N., and I.H., as well as an external expert) hand-coded the fixations in the infant data. A subset of the infant data from Hessels, Hooge, and Kemner (2016) were extracted for manual coding. The data from 20 infants, amounting to a total of 40 min of eye movement data, were coded. Hand coding was done in custom MATLAB software and took each manual coder approximately 3 h to complete.

### Algorithms for comparison

Seven algorithms were chosen for comparison against the I2MC algorithm. Because only three of the seven algorithms provided output for all noise and/or data loss levels, only these algorithms are discussed here. The remaining four algorithms are included in Appendix B. For each algorithm, only parameters that were dependent on the sample frequency of the input data were adjusted. They were set to match their initial values for the 300-Hz data we used here. The search and categorization rules, as well as the motivation for including each algorithm, are described below.

#### Adaptive velocity algorithms for low-frequency data

An implementation of an adaptive velocity search rule is given by Hooge and Camps (2013; hereafter, *HC*). Their algorithm labels fixations instead of saccades and was originally designed for low-frequency (120 Hz or lower) eye movement data. The categorization rules that they employed are (1) adjacent fixations that are less than 1.0° away from each other are merged, and (2) fixations shorter than 60 ms are excluded. This algorithm’s was included because it is a simple fixation-labeling algorithm with few parameters that is based on a threshold that is adaptive to the noise level in the data.

#### Binocular-individual threshold

Another implementation of an adaptive velocity search rule was given by van der Lans, Wedel, and Pieters (2011). They described their algorithm as follows: “Our Binocular-Individual Threshold (BIT) algorithm for identifying fixations is . . . a parameter-free fixation-identification algorithm that automatically identifies task- and individual-specific velocity thresholds by optimally exploiting the statistical properties of the eye-movement data across different eyes and directions of eye movements” (p. 240). The algorithm improves over standard adaptive velocity search rules by using the covariance between movement of the left and right eyes. If the left eye moves in a given direction, the right eye often does so too, whereas in noise the movement of the two eyes is uncorrelated. Given this feature, the BIT algorithm may more readily be able to distinguish saccades from noise than are standard velocity algorithms, and therefore we included it in this comparison. No further categorization rules are reported by the authors for the algorithm.

#### Identification by analysis of variance and covariance

Veneri et al. (2011) designed a fixation-labeling algorithm (hereafter, *C-DT*) with a search rule based on the covariance of the horizontal and vertical eye position signals. The algorithm labels gaze samples as belonging to a fixation when an *F* test indicates that the variances of the horizontal and vertical eye positions are equal. When the covariance between *x*- and *y*-coordinates is high, samples are labeled as belonging to a saccade. The remaining samples are labeled according to a combination of their covariances and the *F* test for equal variances. Veneri et al. reported that their C-DT algorithm identified fixations more accurately than a standard dispersion algorithm when the noise amplitude was high. The reason it was included is that it appears to be robust to noise. No further categorization rules are reported by the authors for the algorithm.

## Results

### RMS noise

**Fig. 3:**
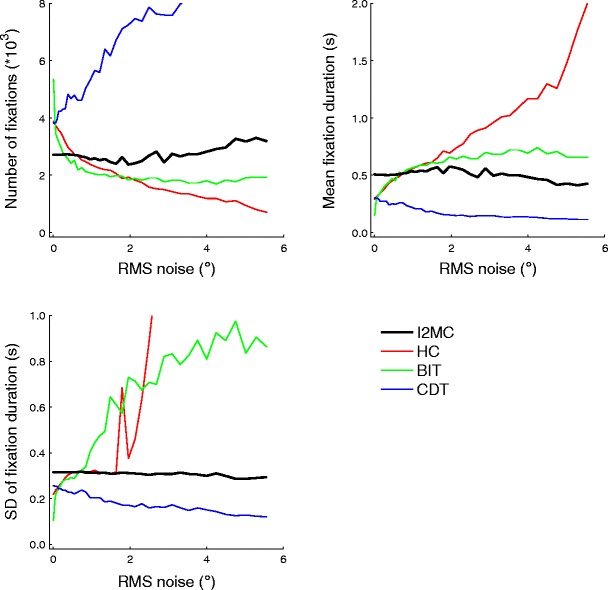
Numbers of fixations (top left), mean fixation durations (top right), and standard deviations of fixation durations (bottom left) for four event detection algorithms as a function of RMS noise added to the eye movement data

The numbers of fixations, mean fixation durations, and standard deviations of the fixation durations were calculated for all algorithms as a function of the RMS noise level added to the eye movement data. As is depicted in Fig. 3, how the algorithms’ outputs were affected by the noise level varied greatly. The HC and BIT algorithms showed immediate decreases in the numbers of fixations detected as noise increased. The number of fixations for the HC algorithm slowly decreased but did not quite reach zero. For the BIT algorithm, the number of fixations stabilized at RMS noise values larger than 2°. The CDT algorithm showed a steady increase in the number of fixations detected as the noise level increased. Finally, the I2MC algorithm produced a fairly consistent number of fixations as a function of noise level, with only a small increase in the total number when more than 5° of RMS noise was added to the eye movement data.

The HC algorithm showed an increase in the mean fixation duration as noise increased, as well as an increase in the standard deviation of the fixation durations. The BIT algorithm also showed increases in both the mean fixation duration and the standard deviation of the fixation duration. The CDT algorithm showed a slowly decreasing, but still fairly consistent, mean fixation duration and standard deviation of the fixation duration. Finally, the I2MC algorithm showed both a fairly consistent mean fixation duration and standard deviation of the fixation duration as a function of noise level, with only a small decrease in the mean fixation duration when more than 5° of RMS noise was added to the eye movement data.

These findings show that the outcome measures from the I2MC algorithm were most robust, among the algorithms we tested, to increasing noise levels in the eye movement data.

### Variable RMS noise

**Fig. 4:**
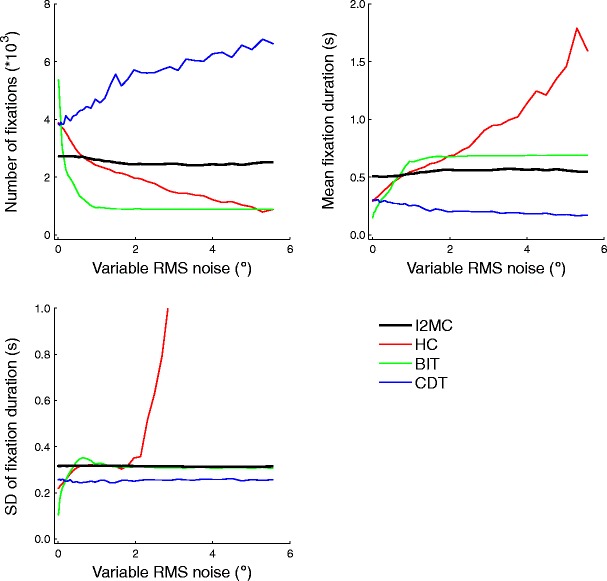
Numbers of fixations (top left), mean fixation durations (top right), and standard deviations of fixation durations (bottom left) for four event detection algorithms as a function of variable RMS noise added to the eye movement data

To determine how robust the algorithms were to large variations in noise level during a single portion of the eye movement data, the RMS noise level was increased only in segments totaling half of the trial. This mimicked short bursts of noise, as may occur, for instance, when tracking is unstable for one part of the screen. The eye movement data in each trial thus contained periods both with and without added RMS noise. As is depicted in Fig. 4, we again observed some variation between the algorithms’ reported numbers of fixations, mean fixation durations, and standard deviations of the fixation durations. The results for the numbers of fixations detected closely resembled those from the earlier RMS noise analyses, although the decreases and increases were smaller. The BIT and HC algorithms showed decreases in the number of fixations, whereas the CDT algorithm showed an increase in the number of fixations. Finally, the I2MC algorithm showed a stable number of fixations as a function of variable RMS noise level.

For the mean fixation durations and the standard deviations of the fixation durations, the results overall matched the previous RMS noise analyses, albeit once again with smaller increases and decreases.

### Data loss

**Fig. 5:**
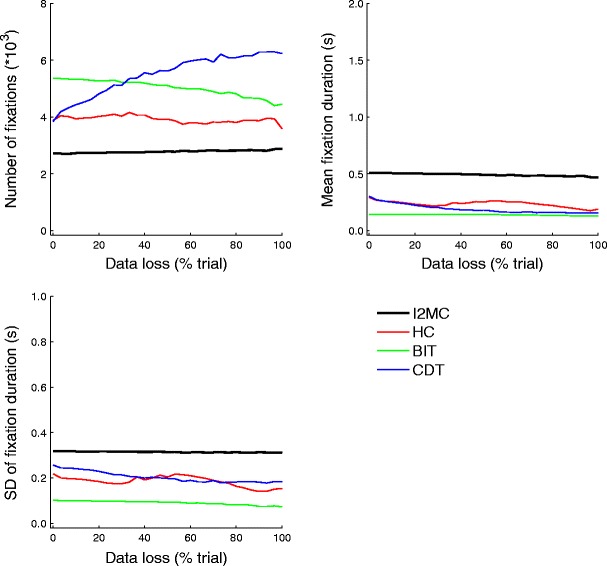
Numbers of fixations (top left), mean fixation durations (top right), and standard deviations of fixation durations (bottom left) for four event detection algorithms as a function of data loss added to the eye movement data

The numbers of fixations, mean fixation durations, and standard deviations of the fixation durations were calculated for all algorithms as a function of the amount of data loss added to the eye movement data, from 0 % (no data loss occurring) to 100 % (data loss could occur throughout an entire trial). As is depicted in Fig. 5, the differences between the algorithms were much smaller than in the previous analyses. The CDT algorithm showed an increase in the number of fixations as data loss increased, but the BIT algorithm, on the other hand, showed a decrease in the number of fixations as data loss increased. Both the HC and I2MC algorithms were stable with regard to the number of fixations as a function of the data loss added to the eye movement data.

For the CDT algorithm, the mean fixation duration and standard deviation of the fixation duration decreased as a function of data loss. For the HC algorithm, a slight decrease was followed by an increase, and then again by a decrease for both the mean fixation duration and the standard deviation of the fixation duration as a function of data loss. Finally, for the BIT and I2MC algorithms, the mean fixation duration was fairly stable as data loss increased.

### Combined noise and data loss

As a final test of the robustness of the outcome measures of the four algorithms, a combination of RMS noise and data loss was added to the eye movement data.

**Fig. 6:**
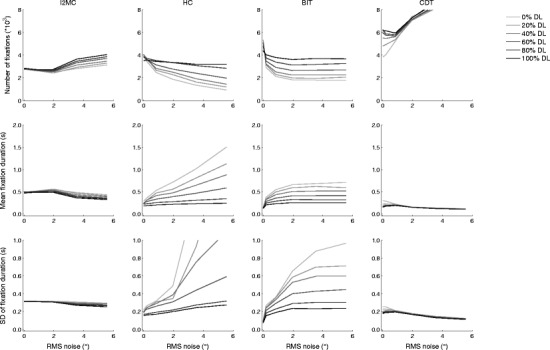
Numbers of fixations (top), mean fixation durations (middle), and standard deviations of fixation durations (bottom) for the final four algorithms as a function of noise level in the eye movement data. From left to right, the columns depict the I2MC, HC, BIT, and CDT algorithms. Separate lines indicate data loss added to 0 % (lightest gray) to 100 % (black) of trials

As we described in the noise analysis, and as is visible in the left column of Fig. 6, there was only a small increase in the number of fixations detected by the I2MC algorithm as the RMS noise increased. When both data loss and noise increased, the increase in the number of fixations detected was somewhat larger than when only the noise level increased. Consequently, the mean fixation duration and the standard deviation of the fixation duration both decreased with increasing noise level, and more so when data loss also increased. For the three competing algorithms (HC, BIT, and CDT), the differences in performance are markedly larger. As is visible in the second column of Fig. 6, the number of fixations decreased for the HC algorithm as the noise level increased. Moreover, the differences between the levels of data loss were large, and the number of detected fixations increased fourfold as data loss increased at the highest noise level. For the HC algorithm, the mean fixation duration and the standard deviation of the fixation duration as a function of noise level increased most for the lowest data loss level, and appeared to be most robust for the highest data loss level (we return to this apparent robustness shortly). As is visible from the third column of Fig. 6, the results for the BIT algorithm are similar to those from the HC algorithm. However, whereas the HC algorithm showed an increasing number of fixations and a decreasing mean fixation duration and standard deviation of the fixation duration across all noise levels, the values for the BIT algorithm appeared to stabilize for RMS noise levels greater than 2°, albeit with marked differences between the levels of data loss. Finally, as is visible in the right column of Fig. 6, the number of detected fixations for the CDT algorithm increased as a function of noise level for all levels of data loss. Consequently, the mean fixation duration and the standard deviation of the fixation duration both decreased as a function of noise level for all levels of data loss. Our conclusion was that the number of fixations, mean fixation duration, and standard deviation of the fixation duration were all most robust for the I2MC algorithm as noise level and/or data loss increased.

### Interpreting noise robustness

**Fig. 7:**
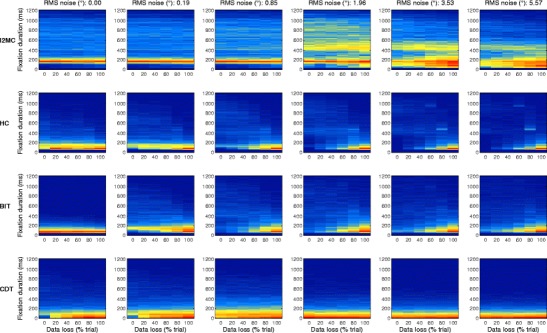
2-D histograms of fixation durations for fixations detected by the I2MC, HC, BIT, and CDT algorithms. The columns depict different noise levels, from low (left) to high (right). Within each histogram, five levels of data loss are depicted, from 0 % (left) to 100 % (right) of the trial. Redder colors indicate more detected fixations, and bluer colors fewer detected fixations

For three of the algorithms, there was at least one data loss level at which the outcome measures were robust to changes in noise level. First, the outcome measures from the I2MC algorithm were the most robust, among all the algorithms, to noise level, data loss level, and the combination thereof. Second, the outcome measures for the BIT algorithm were relatively robust to increases in noise level, albeit with marked differences between the different levels of data loss. Third, the outcome measures reported for the HC algorithm were robust to increases in noise level for the highest data loss level. To interpret this noise robustness, we examined the precise differences in the distributions of fixation durations between these algorithms. Thus, 2-D histograms of fixation duration were computed for the varying noise and data loss levels. As is visible in Fig. 7 (top panels), the distribution of fixation durations detected by the I2MC algorithm remained almost unchanged from 0° to 0.85° RMS noise and from 0 % to 100 % data loss. Note that, because a number of fast corrective saccades followed undershoot of the target in this dataset, there is a large peak in fixation duration around ~140 ms. For higher RMS values, fewer short fixations were reported, and consequently relatively more longer fixations were reported. The differences in the distribution of fixation durations as a function of data loss remain minimal for the 1.96° RMS noise level. For the higher RMS noise levels (3.53° and 5.57°), a larger number of longer fixation durations were reported in general than at the lower RMS noise levels. Moreover, more short fixations were reported at the higher data loss levels for these two RMS noise levels.

**Fig. 8:**
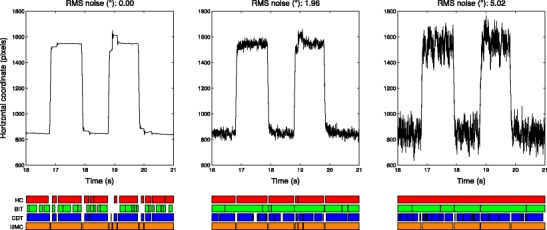
Fixations labeled by the four algorithms for the data prior to adding RMS noise (left), as well as with 1.96° (middle) and 5.02° (right) of added RMS noise. The fixations labeled by the algorithms are presented in the bottom rows. The data are from a trial in which 10° horizontal saccades had to be made

For both the HC and BIT algorithms (middle two rows of Fig. 7), there were few differences in the distributions of fixation durations as data loss increased for the lowest RMS noise level. For RMS noise levels of 0.85° and up, however, both algorithms reported progressively fewer short fixation durations for the lowest data loss level. When data loss increased, progressively more short fixations were again reported. This suggests that both the HC and BIT algorithms detected longer fixations when noise increased, which were subsequently broken up into shorter fixations when data loss increased. Finally, for the CDT algorithm, progressively more short fixations were reported when the RMS noise exceeded 1.96°. The differences between the distributions of fixations durations for the varying data loss levels were minimal, most probably due to the fixation durations already being extremely short. As is visible in Fig. 8, the numbers of fixations and the fixation durations detected by the I2MC algorithm were more noise-invariant than the outputs of the other three algorithms. To conclude, the numbers of fixations and the distributions of fixation durations in the output from the I2MC algorithm were affected least by both noise level and data loss level.

### Applying the algorithm to infant data

**Fig. 9:**
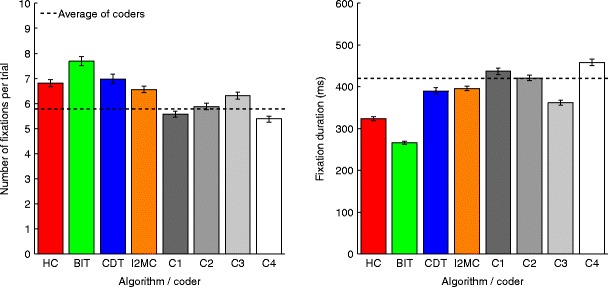
Average numbers of fixations per trial (left) and average fixation durations (right) for infant eye movement data, as reported by the four algorithms and four coders. Error bars represent standard errors of the means, and the dashed lines indicate the averages of the four coders

After ascertaining the noise robustness of the I2MC algorithm, it and the competing algorithms were applied to 40 min of infant data. The average numbers of fixations per trial and the mean fixation durations were calculated for the I2MC, HC, BIT, and CDT algorithms, as well as for the four expert coders. The algorithm that best approached the average of the four expert coders was considered the best in the application to infant data. As is visible in Fig. 9, there was some variability in the outcome measures of the expert coders. Moreover, the expert coders generally detected fewer fixations per trial, and the mean fixation duration was generally longer than with the algorithms. When comparing the algorithms to the average of the four expert coders, a couple of conclusions can be drawn. The HC and BIT algorithms detected approximately one or two fixations more per trial than the average of the expert coders, and the mean fixation duration was between 100 and 150 ms shorter than the average of the expert coders. Both the CDT and I2MC algorithms approximated the average of the expert coders better, and the I2MC did so the best. The differences between the results from the I2MC algorithm and the average of the four expert coders were 0.77 fixations per trial and 24 ms in mean fixation duration.

**Fig. 10:**
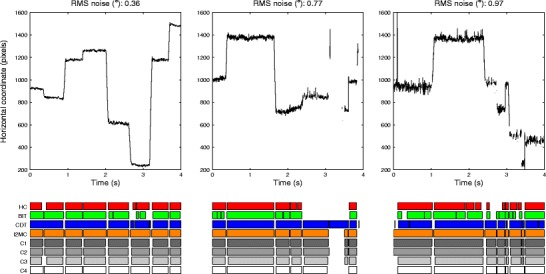
Fixations labeled by the four algorithms and four coders for three representative trials containing infant eye movement data of varying noise levels. Fixations labeled by the algorithms and coders are presented in the bottom rows. Noise levels were estimated by computing the RMS noise of the longest fixation detected by the I2MC algorithm

Three representative excerpts of infant eye movement data with varying noise levels are depicted in Fig. 10. When considering the trial with the lowest noise level (left panel), the numbers and durations of the fixations detected by the four algorithms and the four coders are highly similar. However, for the trial with higher noise levels (middle panel), HC and BIT detected more fixations than did I2MC, CDT, and the expert coders. Finally, for the trial with the highest noise level (right panel), CDT also detected more fixations than the four expert coders. Across the different noise levels, the I2MC algorithm best agreed with the four expert coders. The main difference between the I2MC algorithm and the expert coders was that the fixation durations were slightly longer for the I2MC algorithm. In conclusion, the I2MC algorithm not only performs best in eye movement data with artificially increased noise and data loss levels, but also when applied to actual infant data.

## Discussion

The purpose of the present work was to address the need for an algorithm capable of labeling fixations across a wide range of noise levels in data in which periods of data loss may occur. This is particularly relevant because of the rise of remote video eye-tracking in participant groups such as infants, school children, and certain patient groups whose body movement is difficult to restrain– which may strongly affect the eye movement data quality. Here we proposed and evaluated a new algorithm designed specifically for eye movement data of varying data quality: identification by two-means clustering.

In comparison with other state-of-the-art event detection algorithms, we found the following. First, we report that the numbers of fixations, mean fixation durations, and standard deviations of the fixation durations recorded by the I2MC algorithm were most robust to increases in noise level than the same measures recorded by competing algorithms. This was the case both when the noise level was increased for the entire eye movement signal and when the noise level was increased for only part of the eye movement signal, mimicking short bursts of noise. Second, differences between the algorithms were smaller for the data loss analysis than for the noise analyses. However, the outcome measures of the I2MC and BIT algorithms were most robust to increases in data loss. We report that when adding both noise and data loss to eye movement data, the outcome measures for the I2MC algorithm are most robust. This was particularly the case for 0°–2° of noise, at which levels the outcome measures were almost identical. Since the BIT algorithm appeared to show stable outcome measures as a function of noise for noise amplitudes larger than 2°, albeit with marked differences between the data loss levels, and the HC algorithm showed stable outcome measures for the highest data loss level only, we examined the distributions of fixation duration more closely. Here, we report that the I2MC algorithm showed nearly identical distributions of fixation durations for the lowest noise levels, regardless of data loss level, whereas differences in the distributions of fixation durations for the competing algorithms were already present at the lowest noise levels. However, when the noise amplitude was larger than 2°, the distribution of fixation durations according to the I2MC algorithm also began to show marked differences as compared to the lower noise levels. Finally, when the I2MC was applied to infant data, the outcome measures best approached the average of those of four expert coders, as compared to the other algorithms. We concluded that the outcome measures for the I2MC algorithm were most robust to noise, most notably in the range 0°–2° of RMS noise (for each data loss level) added to the eye movement data.

If we compare the noise robustness of the I2MC algorithm to what has been found in previous research, we see that it also compares favorably to manufacturer-provided algorithms. For instance, Zemblys and Holmqvist (2016) reported that the numbers of fixations and mean fixation durations for the SMI I-VT and I-DT algorithms changed drastically for noise levels higher than 0.25° RMS. In addition, they created a mathematical model to estimate the best-compromise threshold for the SMI dispersion- and velocity-threshold algorithms from the noise levels observed in the eye movement recording. Although adjusting the threshold may compensate for higher noise levels, this comes at the cost of reduced agreement of the algorithms’ output (i.e., numbers of fixations and mean fixation durations) relative to the gold standard. Moreover, a large-scale study of data quality revealed that many of remote eye-trackers produce data with noise levels over 2° for some of participants, with infrequent recordings in which the RMS noise level was even over 3° (Zemblys & Holmqvist, 2016). In addition, Hessels, Kemner, et al. (2015) reported that the RMS noise was rarely over 3° in infant research. The fact that the outcome measures of the I2MC are noise-robust, and particularly so between 0° and 2° RMS noise, means that the algorithm may apply to most real-world situations. Moreover, no parameters need to be adjusted to achieve the same output of the fixation parameters when the noise level varies between 0 and 2°, whereas this was the case for manufacturer-provided algorithms in this range (Zemblys & Holmqvist, 2016).

As we noted in our introduction, a fixation is defined by how it is computed. This means that a fixation for one algorithm is not the same as a fixation for another algorithm. Even when the noise level was low in the eye movement data investigated here, there were large differences between algorithms in the numbers of fixations and the fixation durations (see also Appendix B). This was not only a result of the search rule algorithms apply to find fixation candidates, but also of the categorization rules used to merge or exclude these fixation candidates. When one algorithm excludes more fixation candidates under uncertainty, it will produce fewer fixations as output than will an algorithm that does not exclude any fixation candidates. As such, comparing fixation parameters between algorithms is like comparing apples and oranges. Instead of doing so, we first compared the fixation parameters for each algorithm to itself as a function of data quality—the absolute values for each algorithm were not so important, only the change in the value as a function of noise or data loss. In essence, we compared apples to apples, and oranges to oranges. Here we have presented an algorithm that produces the same output under a wide range of circumstances—the apple remains roughly the same, regardless of the situation. Does this then mean that the output of the presented algorithm is “good”? That is a very difficult question to answer. Commonly in the literature, this question is tackled by comparing the output of an algorithm to an expert coder. Here, when comparing the outcome measures of I2MC and the competing algorithms to the output of four expert coders, I2MC also outperformed the other algorithms. It should be noted, however, that the expert coders did not produce identical outcome measures, such that the question becomes how informative one expert coder actually is. Future research should examine whether expert coders serve as a good gold standard for event detection algorithms.

The I2MC algorithm is applicable to fixation labeling in situations in which the data quality may be low—for instance, when working with infants, school children, or certain patient groups. The I2MC algorithm may also be used when the noise and data loss levels are markedly different between trials and/or participants; the outputs should be comparable despite these differences in noise and data loss levels. This is also particularly relevant for studies in which two groups are compared: For instance, Shic, Chawarska, and Scassellati (2008) reported that changing the parameters of a fixation detection algorithm may reverse the effects between toddlers with autism spectrum disorder and typically developing controls. In addition, recent work has also suggested that differences in data quality between groups should be carefully monitored (Keehn & Joseph, 2016). Here, using an algorithm whose outcome measures are robust to differences in data quality between groups may be a better solution.

Although the I2MC algorithm may be applicable in a wide range of studies, it has several limitations. First, the algorithm is built only for labeling fixations. When saccade parameters are of interest, for example, the I2MC is not a sensible analysis tool. Second, we restricted the present study to labeling fixations in data collected with remote or tower-mounted eye-trackers using static stimuli. When head-mounted eye-trackers are used, the gaze position signal is complicated by periods of optokinetic nystagmus and the vestibulo-ocular reflex, which may require a different event detection strategy. Moreover, when moving stimuli are used in remote or tower-mounted eye-trackers, smooth pursuit movements may occur. Recent work has begun to address event detection in these situations (Larsson et al., 2015). In addition, a general limitation to the present work is that we focused on 300-Hz data, whereas a broad range of sampling frequencies are being used in eye-tracking research. Moreover, although the selection of algorithms against which we tested the I2MC algorithm was motivated, it represents but a subset of the entire event detection catalog.

## Conclusion

Here we presented a fixation detection algorithm for eye-tracking data recorded with remote or tower-mounted eye-trackers using static stimuli. The algorithm works offline and is automatic. The key improvement made by this algorithm is that it labels fixations across a wide range of noise levels and when periods of data loss may occur.

## Figures and Tables

**Fig. A1:**
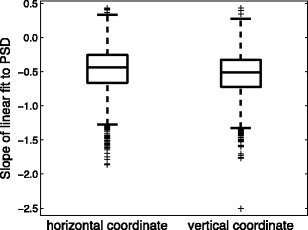
Boxplots for the slopes of the linear fit to the power spectrum density (PSD) for the horizontal and vertical gaze coordinates

**Fig. B1:**
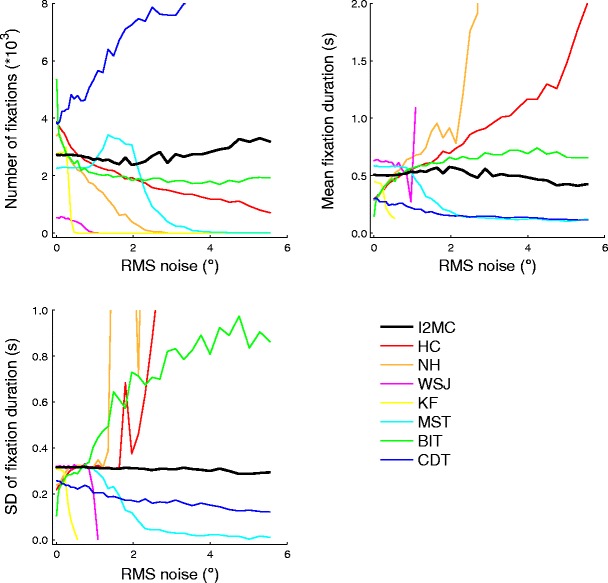
Numbers of fixations (top left), mean fixation durations (top right), and standard deviations of fixation durations (bottom left) for all eight event detection algorithms as a function of RMS noise added to the eye movement data

**Fig. B2:**
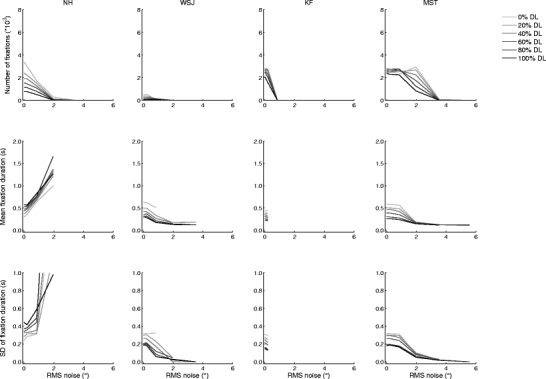
Numbers of fixations (top), mean fixation durations (middle), and standard deviations of fixation durations (bottom) for the four additional algorithms as a function of noise level in the eye movement data. From left to right, the columns depict the NH, WSJ, I-KF, and I-MST algorithms. Separate lines indicate data loss added to from 0 % (lightest gray) to 100 % (black) of a trial

## Appendix A

### Noise

Here we describe how the noise characteristics of the infant eye movement data were determined and re-created. The following steps were performed:

1. We performed event detection on the eye movement data using I2MC (chosen for its event detection over a wide range of noise levels and data loss).
2. For each trial we selected all fixations that were at least 400 ms long.
3. For each of these fixations, we determined the frequency characteristics of the noise separately for the horizontal and vertical eye position signals as follows:
  - We centered and Fourier-transformed the central 400 ms of the fixation using the FFT algorithm in MATLAB R2015b, yielding the frequency domain Fourier coefficients ${C}_k$, where *k* ranges from 0 to 60 for our 400 ms of data at 300 Hz.
  - We then calculated the power spectral density (PSD) using standard techniques. Specifically, using the symmetry of the Fourier coefficients around the Nyquist frequency that is obtained when transforming a real signal, the PSD was obtained as ${C}_k{C_k}^{*}/120*2$.
  - Noise can be characterized by the examining how the PSD changes as a function of frequency. In biological systems, the PSD is often inversely proportional to the frequency, and as such characterized by $1/{f}^{\alpha }$, where *f* is the frequency and *α* is a scaling exponent. Such scaling relationships have also been observed in human eye movements, with scaling exponents between –0.6 and –2 (Coey, Wallot, Richardson, & van Orden, 2012; Findlay, 1971; Wang, Mulvey, Pelz, & Holmqvist, 2016). The *α* value was determined by the slope of a straight line fitted to the PSD in log–log space.
  - The mean slopes of all fixations were –0.48 for the horizontal axis and –0.54 for the vertical axis, which were values comparable to previous results (Coey et al., 2012). However, as is visible in Fig. A1, there is considerable variation in the observed slopes. To ensure that we adequately represented the observed range of slopes in the noise generated below, we cast the fixations into 100 bins (each containing an equal number of data points), based on the slope obtained from the PSD fit. We then determined the mean PSD in each bin by taking the PSDs for all trials in the bin and averaging these.

The distribution of PSDs thus derived was used to generate random noise with sample-to-sample RMS levels between 0° and 5.57°. For each noise level, a noise signal was generated and added to each trial of the dataset with the following procedure:

1. Randomly choose one of the 100 PSDs generated from the infant data set.
2. Interpolate the PSD so that it corresponds to the number of samples for which we want to generate data.
3. Generate a random signal with the same number of samples as the trial, and take its Fourier transform. Combine the phase spectrum of this signal with the interpolated PSD generated in Step 2.
4. Compute the random noise time series with the target PSD by taking the inverse Fourier transform of the combined signal from Step 3.
5. Determine the sample-to-sample RMS of this noise time series and scale the time series to achieve the desired RMS noise level.

### Data loss

We have previously described the empirical distribution of durations of data loss for the baby dataset used here (Hessels, Andersson, et al., 2015). To be able to generate representative data loss, we expanded on this by also describing the distribution of the durations of data between the periods of data loss and the relationship between the duration of one period of data loss and the duration of the following period of data loss. To determine these relationships, we analyzed the data loss in the baby data with the following procedure:

1. For each period of data loss, we computed its duration, the duration of data until the next period of data loss, and the duration of the next period of data loss.
2. We summarized these data as a 3-D histogram indicating the frequencies of co-occurrence of every possible combination of the three variables. Because none of the algorithms deal with loss periods longer than 100 ms, the histogram was truncated to this value for loss duration and the next loss duration.

It should be noted that the procedure above describes what the loss looked like when there was loss; it does not capture how frequently loss occurred overall in the data. For instance, trials in which no loss occurred, as well as the data duration before the first loss period and after the last loss period in a trial, are not represented in our histogram. However, the aim was that the data loss that was present be representative of how data loss occurs in suboptimal recordings. Given the above histogram as input, we sampled data loss and added it to between 0 % and 100 % of a trial, with the following procedure:

1. For the first data loss period, randomly choose a point in the 3-D histogram, taking the relative frequency of occurrence as a probability weighting for each point being selected.
2. Then, for all the next samples, use the duration of the next period of data loss, just given by the previous sample, to determine the duration of the current loss period. Then sample from the subset of the 3-D histogram given by the current data loss duration to determine the duration of data until the next period of data loss and the duration of the next period of data loss, again using the relative frequencies of occurrence of each combination as probability weights.
3. Continue with Step 2 until data loss has been generated for the whole trial. Step 1 is repeated in the very rare case that there are no observations of a given loss duration in the 3-D histogram.

To add data to a specific percentage of a trial, we defined 24 equally spaced time points in the trial. Data loss was added in windows centered on these time points, with the window size set such that the desired amount of loss was attained.

## Appendix B

### Additional algorithms for comparison

#### Adaptive velocity algorithm (NH)

A recent advancement in event detection was introduced by Nyström and Holmqvist (2010). Their search rule first labels periods of data as noise when the velocity exceeds a physiological implausible value. Subsequently, a velocity threshold adapts to the remaining noise level for finding saccade-candidates, which are labeled as saccades if they exceed the minimum saccade duration (i.e., the categorization rule for saccades). Hereafter, periods of data following a saccade are labeled as postsaccade oscillations if they contain a peak exceeding a certain velocity threshold. Finally, the remaining samples are labeled as fixation candidates. If consecutive samples exceed the minimum fixation duration (i.e., the categorization rule for fixations) of 40 ms, they are labeled as a fixation. The reason for its inclusion in the present comparison was that, in our experience, it is one of the best event detection algorithms currently available for low-noise data. We reasoned that, since it employs an adaptive velocity threshold set depending on the noise level in the data, the outcome measures derived from its output might be robust to noise for at least a range of noise levels.

#### Identification by velocity threshold for low- and high-noise data (WSJ)

Wass et al. (2013) adapted a velocity threshold search rule with several post-hoc categorization rules, as we detailed in the introduction. In short, the algorithm uses a fixed velocity threshold to determine fixation candidates, and thereafter excludes those fixation candidates that are unlikely to be genuine fixations according to a set of rules. The reason the algorithm was included here is that it was specifically designed to accomplish fixation labeling in both low- and high-noise data.

#### Identification by Kalman filter (I-KF)

In the I-KF algorithm, a Kalman filter is used to predict the eye velocity of the current sample on the basis of the observed eye velocities of the previous samples in a noise-suppressing manner. In its search rule, the samples for which the observed velocity differs significantly from the predicted velocity (using a chi-square test) are flagged as saccade candidates (Sauter, Martin, Di Renzo, & Vomscheid, 1991; see also Komogortsev & Khan, 2009). The implementation by Komogortsev et al. (2010) was used in the present comparison. Their categorization rules were subsequently: If fixation candidates are separated by a Euclidean distance of less than 0.5° and less than 75 ms in time, fixation candidates are merged. Fixation candidates shorter than 100 ms are excluded.

#### Identification by minimum spanning tree (I-MST)

A minimum spanning tree algorithm aims to connect all 2-D gaze coordinates with line segments in a tree in such a way that the total length of these line segments is minimized (Salvucci & Goldberg, 2000). In an ideal situation, short line segments connect gaze coordinates during a fixation, and longer line segments connect the separate fixations with each other. The reason I-MST is included here is because “The advantage of using an I-MST is the algorithm’s ability to correctly identify fixation points, even when a large part of the signal is missing due to noise” (Komogortsev et al., 2010, p. 2638). The implementation of I-MST used in the present comparison was provided by Komogortsev et al. (2010). Their subsequent categorization rules were: If fixation candidates are separated by a Euclidean distance of less than 0.5° and less than 75 ms in time, fixation candidates are merged. Fixation candidates shorter than 100 ms are excluded.

### Results and conclusions for the additional algorithms

As can be seen in Fig. B1, the outcome measures of the NH, WSJ, I-KF, and I-MST algorithms are not robust to increases in noise level in the eye movement data. As is visible in Fig. B2, this is also the case for combinations of increases in noise and data loss levels. Importantly, the NH, WSJ, and I-KF algorithms report few or no fixations when the noise level is over 2° RMS noise. Moreover, the numbers of fixations, and consequently the mean fixation durations, are not robust to changes in noise level between 0° and 2° of RMS noise. We concluded that the four additional algorithms do not improve over the I2MC and the algorithms already introduced in the main body of the article. Finally, Table B1 depicts the percent changes in the numbers of fixations, mean fixation durations, and standard deviations of the fixation durations for the noise, variable-noise, and data loss analyses for all eight event detection algorithms. The percent changes in the outcome measures of the I2MC algorithm are smallest, and the I2MC algorithm is therefore considered most robust to changes in eye movement data quality.

**Table B1:** Percent changes in numbers of fixations, mean fixation durations, and standard deviations (*SD*s) of the fixation durations after adding noise, variable noise, and data loss for all algorithms

	Percentage Change: Number of Fixations			Percentage Change: Mean Fixation Duration			Percentage Change: *SD* of Fixation Duration		
Algorithm	Noise	Variable Noise	Data Loss	Noise	Variable Noise	Data Loss	Noise	Variable Noise	Data Loss
I2MC	17	–7	6	–16	7	–8	–7	–1	–2
HC	–82	–77	–8	594	447	–35	1,881	1,598	–30
NH	–100	–96	–76	*	–26	91	*	–38	75
WSJ	–100	–87	–94	*	–26	–50	*	–2	–49
KF	–100	–50	25	*	0	–48	*	0	–48
MST	–99	–50	7	–79	–2	–56	–97	0	–42
BIT	–64	–84	–17	360	382	–12	743	199	–29
CDT	144	73	62	–62	–43	–48	–53	0	–29

^*^Because no fixations were detected for the highest noise level, reporting changes in mean fixation duration and the *SD* of fixation duration is not informative: Changes in the means (and *SD*s) of fixation duration may have been positive or negative for the last noise level at which fixation detection was achieved

## Footnotes

1 Although accuracy is also an aspect of data quality, this does not affect the detection of fixations.
